# Covid-19 in Egyptian hemodialysis and kidney transplant children: retrospective analysis of single center experience

**DOI:** 10.1186/s13052-022-01345-z

**Published:** 2022-08-19

**Authors:** Fatina I. Fadel, Samar Sabry, Mohamed A. Abdel Mawla, Rasha Essam Eldin Galal, Doaa M. Salah, Rasha Helmy, Yasmen Ramadan, Wessam Elzayat, May Abdelfattah, Eman Abobakr Abd Alazem

**Affiliations:** 1 Department of Pediatrics, Pediatric Nephrology and Transplantation Units, Kasr Alainy Faculty of Medicine, Cairo University, Cairo, Egypt; 2 Department of Pediatrics, National Research Center, Giza, Egypt; 3 Department of Radiodiagnosis, Kasr Alainy Faculty of Medicine, Cairo University, Cairo, Egypt; 4 Department of Clinical Pathology, Faculty of Medicine, Cairo University, Cairo, Egypt

**Keywords:** Covid-19, Hemodialysis, Hypertension, Kidney Transplantation

## Abstract

**Background:**

Chronic kidney disease stage 5 (CKD 5) populations have peculiar risk for severe Covid-19 infection. Moreover; pediatric data are sparse and lacking. The aim of this study is to report our experience in CKD 5 children treated by hemodialysis (CKD 5D) and CKD 5 children after kidney transplantation (KTR) during one year of Covid-19 pandemic.

**Methods:**

Retrospective analysis of 57 CKD 5 children with Covid-19 like symptoms during 1 year pandemic was performed. A cohort of 19 confirmed patients (13 CKD 5D and 6 KTR) was analyzed in details as regard clinical, laboratory, radiological criteria, management and their short term outcome.

**Results:**

**Conclusion:**

Pediatric patients on regular HD (CKD 5D) are at higher risk and worse outcome of Covid-19 infection than KT recipients (KTR). Pre-existing HTN and shorter duration after KT are potential risk factors. Reversible AGD after KT and CVC related infections in HD patients are additional presenting features of Covid-19 infection.

## Background

It is now known worldwide that the mortality rate of Covid-19 varies from country to country, age, and specific patient groups [1]. Evidence from adult studies indicates that chronic kidney disease (CKD) and dialysis patients are at high risk of Covid-19 infection [2] and poor outcome [2, 3]. Similarly, kidney transplant (KT) recipients were reported to have an increased risk of critical illness, with a mortality rate up to 28% [4] and up to 33% in solid organ transplant (SOT) recipients [5]. Pediatric population had lower rates of Covid-19 associated mortality than adults ranging from 0.7% among the general pediatric population [6] and up to 3.5% in children with coexisting kidney disease [7].

The European Renal Association Covid-19 Database (ERACODA) showed that within dialysis and KT populations the risk conferred by classical risk factors for severe Covid-19 is completely different than in the general population [8]. In dialysis patients with Covid-19 the relative contribution of age to mortality is considerably less than in general population and morbidities as hypertension; coronary artery disease and diabetes do not confer an independent increased risk of mortality [9]. In pediatric KT recipients with Covid-19; management of immunosuppression (IS) and duration of viral shedding are largely unknown. Also, there is very limited information available on the clinical spectrum of the disease in these patients [10].

## Aim of the study

The aim of this study is to report a single center experience of the clinico-laboratory presentation, management and outcomes of Covid-19 infection in children with chronic kidney disease stage 5 (CKD 5) on regular hemodialysis (CKD 5D) and after KT (KTR).

## Patients and methods

### Patients

**Fig. 1:**
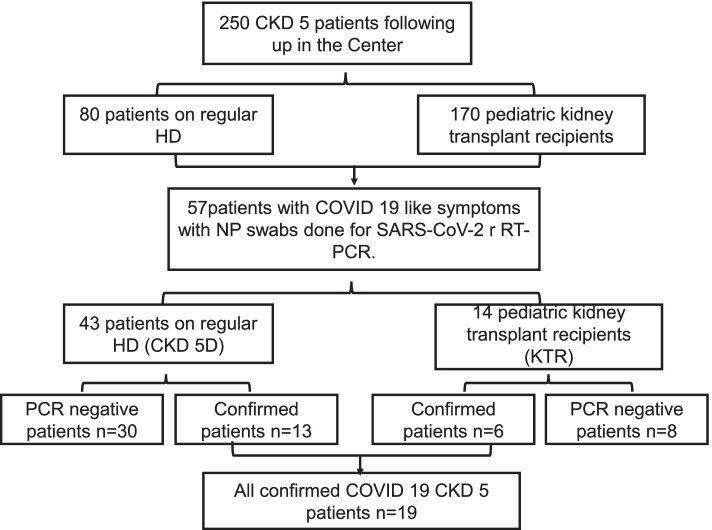
Flow chart illustrates patients enrollment into the study based on Covid-19 symptoms and confirmatory test

This study included essentially fifty seven (CKD 5) pediatric patients presented with Covid-19 like symptoms over one year of Covid-19 pandemic (May, 2020 till May, 2021). Out of included 57 patients; 43 (75.5%) patients were on regular hemodialysis (CKD 5D group) and 14 (24.5%) patients were living donor kidney transplant recipients (KTR group). By the time of conducting this study; a total number of 250 CKD stage 5 patients were following in our Center; 80 patients undergoing regular HD thrice weekly in HD section of Pediatric Nephrology Unit (PNU), Cairo University Children Hospital (CUCH), and 170 pediatric kidney transplant recipients following up at Kidney Transplantation Clinic, CUCH. Out of 57 nasopharyngeal (NP) swabs for Severe Acute Respiratory Virus Syndrome 2 (SARS-CoV-2) real time polymerase chain reaction (RT-PCR) done for all patients with Covid-19 like symptoms; 19 patients (33%) were confirmed cases of Covid-19 infection; (13 patients of CKD 5D group and 6 patients of KTR group) (Fig. 1).

## Methods

This is a retrospective observational study. The study was approved by Pediatric Nephrology Unit, Pediatric Department, Faculty of Medicine, Cairo University. Informed consent was obtained from children care givers prior to inclusion in the study. All procedures followed were in accordance with the Helsinki Declaration of 1964.

Base line patient characteristics were reported as age, gender, original disease, comorbidities, pre-existing hypertension (HTN), dialysis duration, dialysis access, adequacy among the CKD 5D group, and regular treatment of all patients in the CKD 5D and KTR groups. In KTR group graft function was assessed by measurement of serum creatinine and proteinuria. Acute graft dysfunction (AGD) was defined as rise of serum creatinine > 25% of the baseline level [11]. Chronic rejection was defined clinically as a progressive deterioration of graft function with ≥ 15% irreversible rise in creatinine within 1 to 3 months and proteinuria ≥ 1 g/24 h together with a histologic diagnosis of interstitial fibrosis and tubular atrophy (IFTA) [12].

Clinical and epidemiologic information to determine Covid-19 suspicion in the era of pandemic was based on any of the following: a) presence of suggestive symptoms according to CDC (fever and/or acute upper or lower respiratory illness and/or gastrointestinal symptoms), b) patients had close contact with laboratory-confirmed Covid-19 patient within 14 days of symptom onset. c) basic laboratory investigations revealed lymphopenia and elevated acute phase reactant**.**

Specimen from upper respiratory tract (NP) was taken from 57 patients. Under complete safety precautions and full personal protective equipment specimens was collected by a health care professional. NP specimens were collected using a Dacron swab with a plastic shaft by a healthcare provider for each patient [13]. Swabs were placed immediately into a sterile transport cryovial containing 2-3 mL of viral transport medium (VTM) [14]. Specimens from NP were immediately transported in leak proof biohazard bags in an ice box to the PCR laboratory for SARS-CoV-2 rRT-PCR [13, 15]. Follow up NP swabs were taken 10, 28, and 45 days or till 2 successive negative swabs are obtained separated by 48 h interval.

Plain CT scans were obtained at time of admission from all patients using multi-detector CT scanner (Toshiba, Tokyo, Japan; Emotion 16, Siemens, Erlangen, Germany).

The reconstructed CT images (1.25 mm. collimation) are sent to the picture archiving and communication system (PACS) for analysis. The following scores were reproduced: CO-RADS categories and the corresponding level of suspicion for pulmonary involvement in Covid-19 (Table 1) [16] and CT severity score (range 0–25) that was defined as the sum of lung involvement (0: 0%, 1: < 5%, 2: 5–25%, 3: 25–50%, 4: 50–75%, 5: > 75%) of each lobe and a score 0–7 was considered mild, 8–16 moderate, and 17–25 advanced [17].

**Table 1:** CO-RADS categories and level of suspicion for pulmonary involvement

**CO-RADS Category**	**Level of Suspicion for Pulmonary involvement of COVID-19**	**Summary**
0	Not interpretable	Scan technically insufficient for assigning a score
1	Very low	Normal or noninfectious
2	Low	Diagnosing other chest infections not COVID-19
3	Equivocal/unsure	Signs confirming COVID-19 but also present in other diseases
4	High	Suspicious for COVID-19
5	Very high	Typical for COVID-19
6	Proven	RT-PCR positive for SARS-CoV

### Statistical analysis

Statistical Package for Social Sciences version 15 (SPSS, Chicago, Ill) was used to analyze collected data. Nominal data were expressed as frequencies and percentage, parametric data as means and standard deviations (SD). Non parametric data expressed as median and inter quartile range. Nominal data were compared using chi-square test; while numerical data were compared using t- test.Non parametric data were compared using Mann–Whitney (u test).* P* values < 0.05 considered significant.

## Results

This study reports an incidence of Covid-19 symptoms in symptomatic and close contacts HD patients (CKD 5D) and KTRs following up at CUCH over 1 year of pandemic of 16.2% (13/80) and 3.5% (6 /170) respectively. Figure 1 is a flow chart illustrates patients enrollment into the study based on Covid-19 symptoms and confirmatory test.

Treatment of CKD 5 patients on regular HD (CKD5D) was in the form of dialysis therapy and medications: **a**) dialysis was performed using conventional low flux HD machines with polysulfone hollow fiber dialyzers and bicarbonate-containing dialysate three times per week. **b**) medications were in the form of subcutaneous erythropoietin to maintain their hemoglobin above 10 gm% and hematocrit of 35–40%, oral iron therapy, oral vitamin D to maintain acceptable serum calcium, phosphorus/ parathormone levels and medications to control hypertension (mainly in the form of ACE inhibitors and Ca channel blockers). Treatment of CKD 5 patients after KT (KTR) was mainly in the form of classic triple therapy of maintenance immunosuppression protocol that consists of steroids, CNI (cyclosporine or tacrolimus) and adjuvant therapy (MMF or enteric coated mycofenolic acid). No one of included patients received Covid-19 vaccine since it has been approved in Egypt after this study had been conducted.

### Base line and clinical data of Covid-19 confirmed cohort (*n* = 19)

The mean age of Covid-19 cohort is 9.1 ± 4.68 years with male to female ratio of 1.7 (12:7). Demographic, clinical, disease related, dialysis and/or transplantation related data are demonstrated by Table 2.

**Fig. 2:**
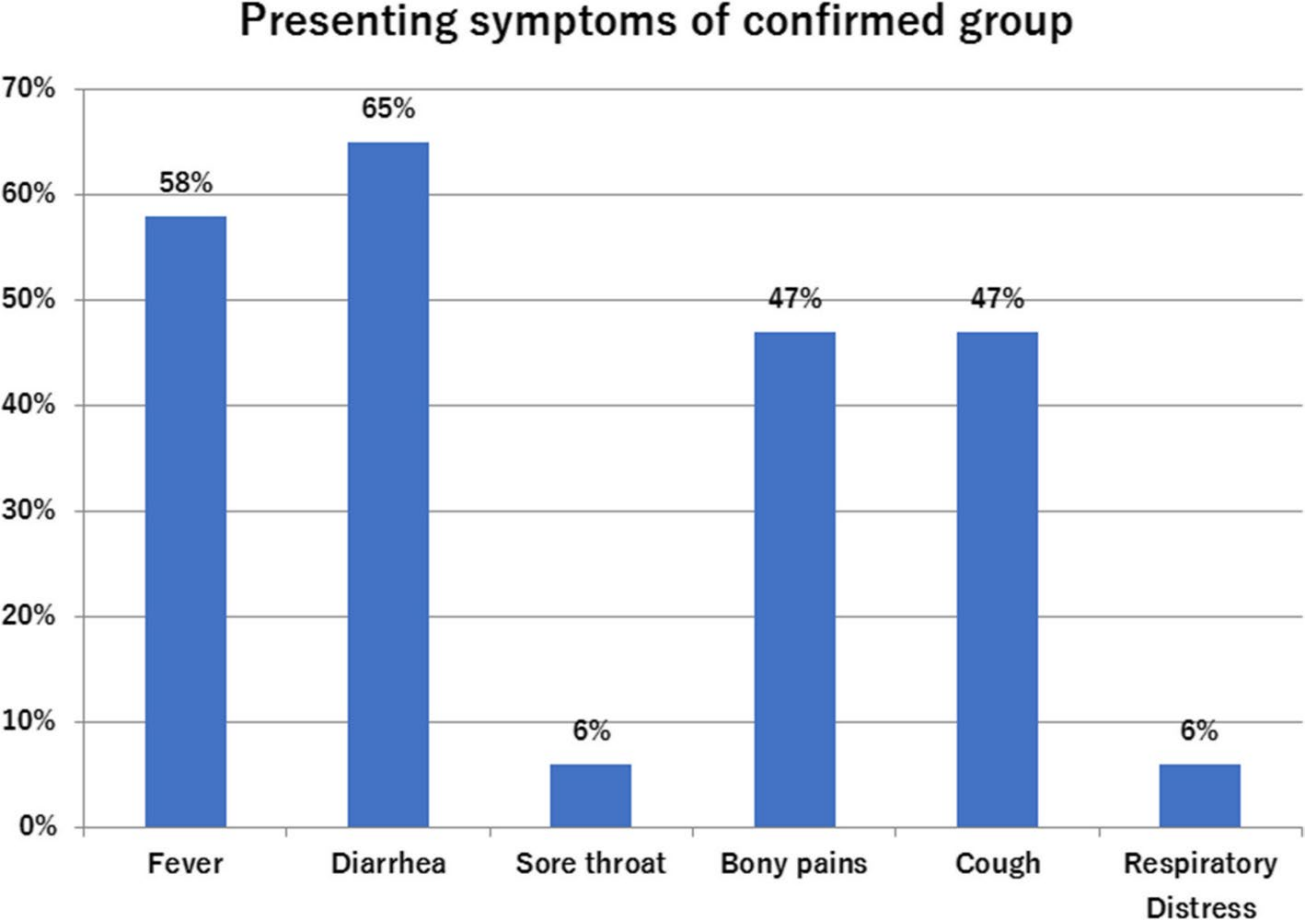
Column chart of presenting symptoms of confirmed cohort (*n* = 19)

Diarrhea was the main presenting symptoms among confirmed cases (68%, *n* = 13), followed by fever (63%, *n* = 12) then respiratory symptoms and bony pains in (52%, 47% respectively). Respiratory symptoms varied from mild upper respiratory tract (URT) symptoms in 7 (36%) patients to severe Covid-19 pneumonia necessitating ICU admission and mechanical ventilation in 3 (15.7%) patients (Fig. 2). Notably; AGD was an associated symptom of 83.3% of KTR group (*n* = 6). Central venous catheter (CVC) related infection was the only presenting feature with fever in 4 patients (30%) of CKD 5D group (*n* = 13).

**Table 2:** Baseline data of Covid-19 NP swab positive and negative patients

		**Covid-19 positive patients (** ***n*** ** = 19)**	**Covid-19 negative patients (** ***n*** ** = 38)**	***p-*** **value**
**Age (years)**	*Mean ± SD*	*8.1 ± 4.512*	*6.6 ± 3.41587*	*0.1658*
**Male/female**	Ratio	12/7	20/18	0.45025
**Original renal disease:**				
FSGS	N (%)	4 (21)	8 (21)	1
NPHP	N (%)	2 (10.67)	4 (10.5)	1
CIN	N (%)	2 (10.67)	6 (15.8)	0.58
VUR	N (%)	5 (26)	9 (23.7)	0.827
Bilateral atrophic	N (%)	4 (21)	8 (21)	1
kidneys	N (%)	2 (10.67)	3 (7.9)	0.741
#Others				
**Hypertension**	N (%)	15 (78.9)	14 (36.8)	**0.0028**
**CKD 5D group**		**Covid-19 positive patients (** ***n*** ** = 13)**	**Covid-19 negative patients (** ***n*** ** = 30)**	
**Dialysis duration (months)**	Mean ± SD	14 ± 6.96	19.7 ± 9.87	0.0664
**Weight (kg)**	Mean ± SD	17.54 ± 7.74	20.12 ± 9.546	0.3881
**Residency (Urban / Rural)**	Ratio	7/6	14/16	0.6653
**Dialysis access (CVL / AVF)**	Ratio	6/7	21/9	0.1337
**Dialysis adequacy (Kt/v)**	Mean ± SD	1.56 ± 0.45	1.61 ± 0.754	0.825
**$Comorbidity**	N (%)	3 (15.8)	9 (23.7)	0.642
**KTR group**		**Covid-19 positive patients (** ***n*** ** = 6)**	**Covid-19 negative patients (** ***n*** ** = 8)**	
**Post-TX follow up duration (mo)**	Mean ± SD	9.5 ± 3.69	19.4 ± 8.54	**0.0215**
**Weight (kg)**	Mean ± SD	25.4 ± 11.23	31.1 ± 15.1	0.4531
**Baseline serum creatinine (mg/dl)**	Mean ± SD	0.95 ± 0.303	0.99 ± 0.45	0.7145
*Trough level of CNI (ng/dl)	Mean ± SD	7.58 ± 3.54	5.9 ± 3.1	0.3631
*Daily steroid dose (mg/day)	Mean ± SD	5 ± 2.25	7.5 ± 3.65	0.176
*Mycofenolate dose (mg/day)	Mean ± SD	584 ± 210	612 ± 325	0.8576

*FSGS*, Focal segmental glomerulosclerosis, *NPHP*, Nephronopthesis, *CIN*, Chronic interstial nephritis, *VUR* Vesicoureteric reflux, *CVL*, Central venous line, *AVF*, Arteriovenous fistula, *BMI*, Body mass index, *TX* Transplantation, *CNI* Calcinurine inhibitors, *HD* Hemodialysis, *KTR* kidney transplant recipients

^#^other original diseases includes (two patients with ARPKD (Autosomal Recessive Polycystic Kidney disease), two patients with hemolytic uremic syndrome and one patient was Alport syndrome)

^$^comorbidity include:chronic lung disease in 10 patients(nine of them were covid negative and their chest condition made them suspected as Covid-19,heart failure in three patients

*Concomitant to time of Covid-19 infection

### Clinical course of the disease in Covid-19 confirmed cohort

Eleven (57.9**%)** patients (10 of CKD 5D group and one of KTR group) had mild symptoms and managed on outpatient basis. Of them ten patients (90%) were symptoms free after a mean duration of 3.8 ± 2.5 days and NP swab turned negative after a mean duration of 19 ± 9.6 days, while one patient deteriorated clinically and needed hospitalization.

Five (26.3%) patients (4 of CKD 5D group and one of KTR group) experienced severe symptoms in term of Covid-19 pneumonia at initial presentation (3 patients needed ICU admission and mechanical ventilation and 2 patients needed only oxygen support). The rest three (15.8%) patients (KTR group only) presented with severe diarrhea and dehydration and one of them was shocked and managed at inpatient unit with improvement of clinical symptoms after mean duration 4 days.

### Laboratory and radiological findings in Covid-19 confirmed cohort

Table 3 demonstrates the main laboratory findings among Covid-19 patients. Lymphopenia, neutrophilia and elevated acute phase reactants were almost universal in all confirmed cases. Chest CT scan examination revealed pulmonary involvement in all confirmed patients except for 2 patients (of KTR group). Pulmonary involvement as evident by CT score did not correlate with clinical findings, 7 (36%) of Covid-19 confirmed cohort had no respiratory symptoms in spite of their CT findings. Overall; CT scan findings revealed moderate lung affection with mean severity score 8.8 ± 4.7 (Table 3).

**Table 3:** Laboratory and radiological data of Covid-19 NP swab positive and negative patients

**Laboratory findings**	**Positive patients** **(*n* = 19)** Mean ± SD	**Negative patients (*n* = 38)** Mean ± SD	***P*** **value**
**CRP(mg/l)**	38.25 ± 14.214	69.458 ± 35.201	**0.0005**
**TLC 10**^**3**^**/ul**	8.125 ± 4.987	13.1 ± 7.701	**0.0134**
**Lymphocytes/ul**	854 ± 320	1320 ± 507.6	**0.0006**
**Neutrophils/ul**	7984 ± 4120.258	9852 ± 4123.012	0.1125
**Hemoglobin (gm /dl)**	11.1 ± 6.2	8.12 ± 3.21	**0.0195**
**D-dimer**	1725 ± 852	––-	–––
**S. albumin (CKD 5D group n = 40)**	4.12 ± 1.985	4.01 ± 2.101	0.85
**CT chest radiological finding**			
Severity score	8.89 ± 4.78	4.01 ± 1.9	**< 0.0001**
Co rads	4.1 ± 2.1	2.6 ± 1.5	**0.0030**

### Received medications and outcome of Covid-19 confirmed cohort

Modification of IS among KTR group (*n* = 6) was in the form of withholding MMF for at least 10 days, keeping CNI at the lowest acceptable trough level and temporary elevation of daily oral steroid dose to 15 mg. Three patients received Hydroquinone with routine ECG before starting administration. Anticoagulation was administrated in all patients and was in the form of enoxaparin (Table 4).

**Table 4:** Medications given for Covid-19 confirmed cohort (*n* = 19)

	**Number (%) of patients**	**Mean ± SD duration (days)**
Hydroquinone	15 (78.9)	6 ± 3.147
Azithromycin	19 (100)	3 ± 1.245
Anticoagulation	19 (100)	10 ± 4.941
Ivermictin	2 (10.67)	7 ± 3.012
*Others	17 (89.5)	5 ± 1.025

^*^Others include:paracetamol,vitamin C, zinc,lactoferrin and vancomycin only in four patients due to concomittant CVL infection

**Fig. 3:**
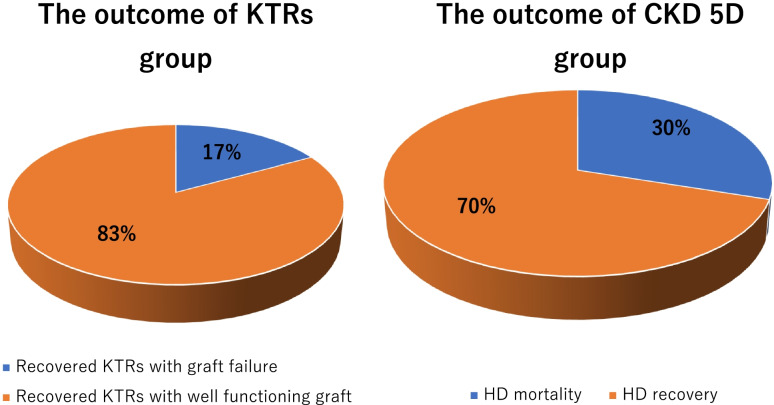
Pie chart describing clinical outcome of confirmed cohort (*n* = 19)

Four out of five patients (80%) of KTR group with AGD had their graft function recovered completely after a median duration of 7 days. One patient had acute on top of chronic ABMR with deteriorating graft function ended up to graft failure 5 weeks after infection. Patient mortality was reported only among CKD 5D group; three patients among CKD 5D group (15.7%) died due to acute respiratory distress syndrome (ARDS) after mean duration of 17 days from developing Covid-19 symptoms, two of them were completely free of symptoms 5 days before developing ARDS (Fig. 3).

### Comparison between symptomatizing Covid-19 negative and Covid-19 positive patients

Covid-19 infection was significantly associated with pre-existing hypertension (HTN) (*p* = 0.002) and lymphopenia (*p* = 0.0006). Covid-19 positive group associated with significant earlier post-transplant duration in KTR (*p* = 0.0215). Pulmonary affection was more severe among Covid-19 positive patients according to CT severity score and CORADs with *p* < 0.0001 and 0.003 respectively (Tables 2, 3).

## Discussion

This study represents a retrospective analysis of a cohort of 19 pediatric CKD stage 5 patients with confirmed Covid-19 infection over 1 year of pandemic [13 patients on regular HD (CKD 5D) and 6 KTRs]. We reported Covid-19 like symptoms incidence of 53.8% (43/80) and 8.23% (14/170) and confirmed Covid-19 infection incidence of 16.2% (13/80) and 3.5% (6 /170) among symptomatic and close contact CKD 5D and KTR patients respectively who are following up in CUCH during the study period.

Our incidence of the diseases is more than what was reported in adult (2.9% in HD patients and 1.4% in KTRs) by ERA-EDTA registry at the beginning of pandemic [18] and by meta-analysis (7.7%) evaluated 29 international studies on adult HD patients [19]. Pediatric studies reported fewer incidences among CKD 5 dialysis and transplanted children (0.6%) during the last year [20, 21]. More recently; Canpolat et al., reported a similar incidence of 9.3% and 9.2% among HD and transplanted Turkish children over nine months of Covid-19 pandemic. Differences in testing strategies between centers and the presence of many asymptomatic cases are challenges to determine the exact incidence of the disease [1]. Our notable less incidence in KTR patients (3.5%) in comparison to HD children (16.2%); is attributed to the fact that caregiver of KTRs are more concerned with isolation as they were instructed from their treating doctors, while HD patients are more exposed to infection as they have to attend hospital by collective transport at least thrice weekly for HD sessions.

Moreover; we observed more prevalence of sever respiratory symptoms among regular HD patients (4/13: 31%) than KTRs (1/6; 16.7%). This observation has more than one explanation; the impact of IS drugs received by patients after KT that possibly blunt cytokine release and control that state of hyper-inflammation present in Covid-19 and eventually leads to absence or less severe respiratory symptoms. Additionally; the strict infection control precautions that is followed by KTRs, as advised by their health care providers, could largely minimize viral load that they exposed to when compared to HD patients.

In the present study we reported AGD as one of the very common clinical presentations in Covid-19 confirmed KTR (83.3%; 5/6). Study on adult KTR revealed 66.6% of cases had AGD on presentation with Covid-19 infection [22]. SARS‐CoV‐2 can cause acute kidney injury (AKI) either by direct invasion of the podocytes and proximal convoluted tubules, also it can cause acute tubular necrosis and protein leakage through angiotensin-converting enzyme 2 (ACE2) pathway and cytokines overproduction lead to renal endothelial cell damage [23]. Moreover; AKI in Covid-19 patients could be aggravated by the state of dehydration, toxic tubular damage, and drug-induced nephrotoxicity [24].

In this study we reported 4 Covid-19 confirmed CKD 5D children presented primarily by CVC infection (exit site infection in addition to blood bacteremia with MRSA) in addition to fever. Covid-19 was reported to be associated with increased risk of bacterial infection including MRSA that is usually associated with high risk of mortality with the use of steroids and empirical antibiotics are risk factors [25]. However this was not the case in our cohort; since our HD patients with CVC infection were accidentally discovered to have concomitant Covid-19 infection during screening with no Covid-19 symptoms apart from fever. Perhaps CVC related and Covid-19 infections reflect similar poor hygienic precautions that a person with CVC related infection is more risky to Covid-19 infection than other HD patients. This hypothesis has been confirmed by Heidempergher study which reported that implementation of hygienic precautions in the dialysis setting can markedly improve the problem of CVC-related infections [26].

Twenty-nine hypertensive patients were reported in this study. Of them, 23 were received ACE inhibitors as their antihypertensive medication. Eleven out of 23 patients using ACE inhibitors developed Covid -19 infection, which was confirmed by PCR. We noticed that Covid-19 infection was significantly associated with pre-existing HTN (*p* = 0.002). Pre-existing HTN was reported to present in up to is 32.7% amongst adult Covid-19 infected patients [27]. In children; a systemic review published by Rodriguez-Gonzale**z** and his colleagues revealed that; cardiovascular involvement seems to be a relevant factor of SARS-CoV-2 infection. They reported that patients with pre-existing cardiovascular diseases constitute a high-risk population for development of a severe acute Covid-19 infection [28]. Moreover; without pre-existing systemic HTN; SARS-CoV induces down regulation of ACE2 in host cells, resulting in increased concentration of angiotensin II which in turn causes severe acute lung injury, also ACE2 has important role in regulation of rennin angiotensin system which is responsible for regulation of blood pressure, vascular resistance and fluid electrolyte balance [29]. This means that relation between HTN and Covid-19 infection is mutual and each can worth the outcome of the other.

We also reported significant association between Covid-19 infection and shorter post-transplantation duration (*p* = 0.0215). This observation is mostly owed to the fact that early post-transplant period is the time of maximization of IS drugs that affect both humoral and cellular immunity leading to increased susceptibility to viral infections including Covid-19 [30].

Diarrhea was reported to be the presenting symptoms of 20–50% of Covid-19 cases [31]. In this study we found that diarrhea was the main presenting symptoms among confirmed cohort (68%), followed by fever (63%) then respiratory symptoms and bony pains in (52% and 47% respectively). Similar results were reported by other studies either in previously healthy [32, 33] or renal [34] children. In a similar study; conducted included 51 hospitalized adult patients with kidney disease including KTR and CKD 5 patients on regular HD; the authors reported fever and cough (55% and 64% respectively) as the main presenting symptoms followed by diarrhea (28%) [35].

Similar to previous reports; lymphopenia, neutrophilia and elevated acute phase reactants were almost universal in all confirmed cases of the present study. Although normal leucocytic count in 74% was reported in a large cohort (*n* = 2597) of previously normal Covid-19 infected children [36], leucopenia and lymphopenia were reported in 31.5% and 9% respectively in pediatrics solid organ transplant recipients [33].

The short term outcome of our confirmed Covid-19 cohort was challenging. Patient mortality was reported only among our CKD 5D group (15.7%) due to ARDS and cytokine storm as release of inflammatory mediators particularly IL6 lead to intravascular coagulation and multiple organ injury with vascular hyperpermeabilty that cause severe systemic inflammation [37]. Accordingly our zero mortality among KTR group possibly as IS drugs blunt cytokine release and control that state of hyper-inflammation present in Covid-19 [38].

To the best of our knowledge; this is the first pediatric report that was conducted over the three waves of Covid-19 pandemic among CKD 5 patients (HD and KTR). This study is limited mainly by being a single center with limited number of patients and by lack of control group of non- renal Covid-19 infected children to outline the impact of renal morbidity on patient's outcome. Further studies are recommended to overcome these limitations.

## In conclusion

Incidence of Covid-19 infection among pediatric CKD 5 children is more in HD patients (CKD 5D) than KTRs.Pre-existing HTN and shorter post-transplantation follow up duration are potential risk factors for Covid-19 infections. Reversible AGD in KTRs and blood stream CVC related infections in HD patients are additional presenting features to Covid-19 infection. Covid-19 infected KTRs have better outcome than HD infected children.

## Data Availability

The data will be available upon request and approval of all authors.

## Abbreviations

ACE2: Angiotensin-converting enzyme 2
AGD: Acute graft dysfunction
AKI: Acute kidney injury
ARDS: Acute respiratory distress syndrome
AVF: Arteriovenous fistula
BMI: Body mass index
CIN: Chronic interstial nephritis
CKD 5: Chronic kidney disease stage 5
CKD 5D: CKD 5 children treated by hemodialysis
CKD: Chronic kidney disease
CNI: Calcinurine inhibitors
CUCH: Cairo University Children Hospital
CVC: Central venous catheter
CVL: Central venous line
ERACODA: The European Renal Association Covid-19 Database
FSGS: Focal segmental glomerulosclerosis)
HD: Hemodialysis
HTN: Hypertension
IFTA: Interstitial fibrosis and tubular atrophy
IS: Immunosuppression
KT: Kidney transplantation
KTR: Kidney transplant recipient
MRSA: Methicillin-resistant Staphylococcus aureus
NP: Nasopharyngeal
NPHP: Nephronopthesis
PNU: Pediatric Nephrology Unit
RT-PCR: Real time polymerase chain reaction
SARS-CoV-2: Severe Acute Respiratory Virus Syndrome 2
SOT: Solid organ transplant
TX: Transplantation
URT: Upper respiratory tract
VTM: Viral transport medium
VUR: Vesicoureteric reflux
